# Prevalence of Circulating Autoantibodies Against G-Protein-Coupled Receptors as Potential Biomarkers for Long COVID: Preliminary Investigations

**DOI:** 10.3390/ijms27041787

**Published:** 2026-02-13

**Authors:** Marta Camici, Marta Franco, Lorenzo Talamanca, Jessica Paulicelli, Liliana Scarnecchia, Manuela Petino, Valentina Mazzotta, Ilaria Mastrorosa, Eleonora Cimini, Eleonora Tartaglia, Stefania Notari, Paolo Zuppi, Roberto Baldelli, Maria Grazia Bocci, Fabrizio Maggi, Enrico Girardi, Andrea Antinori

**Affiliations:** 1 Clinical and Research Infectious Diseases Department, National Institute for Infectious Diseases Lazzaro Spallanzani IRCCS, 00149 Rome, Italy; marta.camici@inmi.it (M.C.); jessica.paulicelli@inmi.it (J.P.); valentina.mazzotta@inmi.it (V.M.); ilaria.mastrorosa@inmi.it (I.M.); andrea.antinori@inmi.it (A.A.); 2 Endocrinology Clinical Unit, San Camillo Forlanini Hospital, 00149 Rome, Italy; martafranco220@gmail.com; 3 Institute of Molecular Systems Biology, Department of Biology, ETH Zürich, 8093 Zurich, Switzerland; talamanca@imsb.biol.ethz.ch; 4 Department of Clinical Pathology, San Camillo Forlanini Hospital, 00149 Rome, Italy; liliana.scarnecchia@gmail.com (L.S.); rbaldelli@scamilloforlanini.rm.it (R.B.); 5 Azienda Sanitaria Locale ASL Roma 1, 00193 Rome, Italy; petinomanuela@gmail.com; 6 Laboratory of Cellular Immunology and Pharmacology, National Institute for Infectious Diseases Lazzaro Spallanzani IRCCS, 00149 Rome, Italy; stefania.notari@inmi.it; 7 Laboratory of Virology, National Institute for Infectious Diseases Lazzaro Spallanzani IRCCS, 00149 Rome, Italy; eleonora.tartaglia@inmi.it (E.T.); fabrizio.maggi@inmi.it (F.M.); 8 Ordine di Malta Italia, San Giovanni Battista Hospital, Endocrinology Project, 00148 Rome, Italy; paolozuppi@libero.it; 9 Intensive Care and Anestesiology, National Institute for Infectious Diseases Lazzaro Spallanzani IRCCS, 00149 Rome, Italy; mariagrazia.bocci@inmi.it; 10 Scientific Direction, National Institute for Infectious Diseases Lazzaro Spallanzani IRCCS, 00149 Rome, Italy; enrico.girardi@inmi.it

**Keywords:** Long COVID, auto-Abs targeting G protein–coupled receptors (GPCRs), IVIG, immunoadsorption, β2-adrenergic-Ab, β2-adrenergic-R, autoimmune signature

## Abstract

This prospective, single-center, case-control study investigated circulating autoantibodies (AAbs) targeting G protein-coupled receptors (GPCRs) in Long COVID (LC) patients to identify potential diagnostic biomarkers and therapeutic targets. Fifteen participants were enrolled at the LC clinic in Rome: eleven with severe LC—defined as >4 persistent symptoms (fatigue, cognitive impairment, poor exercise tolerance, dyspnea, arthralgia, or dysautonomic manifestations) >3 months post-infection—and four asymptomatic post-COVID (APC) individuals. Fatigue was assessed using the Fatigue Assessment Scale (FAS ≥ 22; severe ≥ 35). Auto-Abs against AT1R, endothelin receptor A, adrenergic (α1, α2, β1, β2), and muscarinic (M1–M5) receptors were quantified, along with blood cortisol and ACTH levels. SARS-CoV-2-specific T-cell responses to Spike and Nucleocapsid proteins were evaluated by ELISpot assay. In our small cohort, LC patients were younger, had fewer comorbidities (*p* = 0.03), fewer vaccine doses (*p* = 0.03), and higher FAS scores (33 vs. 12; *p* = 0.001). Mean GPCR AAbs levels were higher in LC than in APC (8.88 vs. 5.45 Units/mL; *p* = 0.17), indicating a coherent autoimmune signature in LC that correlates with symptom development. Morning cortisol was lower in LC (12.7 vs. 17 mg/dL; *p* = 0.01), and T-cell responses tended to be weaker. These findings suggest GPCR AAbs may serve as biomarkers and therapeutic targets for a subset of patients, guiding diagnosis and treatments with IV immunoglobulin or immunoadsorption.

## 1. Introduction

Long COVID (LC) is a multisystemic post-viral syndrome that can persist for months or even years after the acute SARS-CoV-2 infection [1]. It comprises heterogeneous clinical subsets, and it fits within the broader category of the infection-associated chronic conditions sharing numerous clinical and pathophysiological features with Myalgic Encephalomyelitis/Chronic Fatigue Syndrome (ME/CFS) [2]. Although increasing evidence supports underlying biological abnormalities, its pathogenesis remains incompletely understood, and no approved diagnostic biomarkers or established effective treatments are currently available [3]. In light of the condition’s high global prevalence [4], its projected rise in incidence, its profound impact on patients’ quality of life [5], and the considerable social and healthcare burden it represents, the scientific community is intensifying efforts to rapidly close existing knowledge gaps. Emerging evidence suggests that the transfer of immunoglobulin G (IgG) from patients with LC can induce symptoms in animal models, indicating a potential autoimmune or immunopathogenic basis for the condition [6]. Among the proposed mechanisms, particular attention has been given to autoantibodies (AAbs) targeting G-protein-coupled receptors (GPCRs), especially those involved in neurotransmission and vascular regulation [7,8]. Notably, GPCR AAbs directed against β1- and β2-adrenergic receptors, as well as M3 and M4 acetylcholine receptors, have been implicated in both LC and ME/CFS [9,10]. In particular, anti-β2 antibodies appear to be strongly associated with symptom development in both conditions [8]. The primary hypothesis is that these autoantibodies exert a steric inhibitory effect on the receptor, thereby preventing the neurotransmitter from fulfilling its physiological function [10]. Based on these observations, increasing evidence supports the potential use of immunoadsorption [11] and intravenous immunoglobulin (IVIG) [12] as therapeutic strategies for LC and COVID-19-related neurological disorders. One study on immunoadsorption in LC reported that clinical improvement was associated with a reduction in circulating autoantibody levels [11].

We propose that GPCR-directed autoantibodies play a central role in Long COVID pathogenesis by driving dysautonomic manifestations and disrupting the vagal anti-inflammatory reflex as well as vascular and blood flow regulation [10,13].

These mechanisms may, in turn, promote systemic inflammation, endothelial and procoagulant activation, secondary mitochondrial dysfunction [13], and impaired serotonin reuptake at the intestinal level [3].

We present a sub-analysis of a larger cohort study conducted on 15 participants [13]. This exploratory and hypothesis-generating study aims to investigate the prevalence of circulating AAbs targeting GPCRs in participants after the acute SARS-CoV-2 infection, and to evaluate their correlations with clinical symptoms in order to assess their potential as diagnostic biomarkers and therapeutic targets. Furthermore, we evaluated hypothalamic–pituitary–adrenal (HPA) axis function and SARS-CoV-2-specific T-cell responses to gain a more comprehensive understanding of the immuno-endocrine landscape associated with LC. Lastly, we sought to assess a possible AAb-based diagnostic tool.

## 2. Results

**Figure 1:**
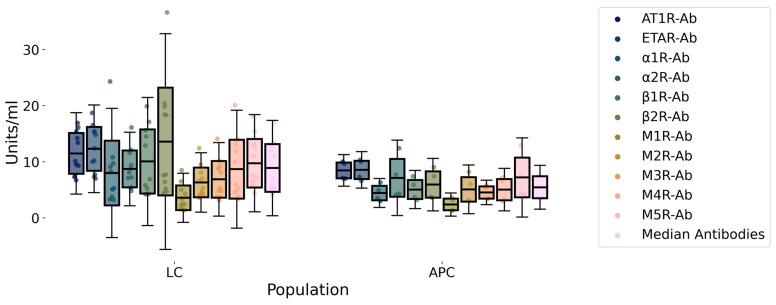
Levels of autoantibodies targeting G-protein-coupled receptors in Long COVID (LC) and asymptomatic post-COVID (APC) patients. Autoantibodies were measured in serum samples from both groups (LC and APC) using ELISA (CellTrend GmbH, Luckenwalde, Germany). Asymptomatic Post-COVID, LC: LongCOVID. AT1R-Ab: anti-Angiotensin II type 1 receptor-Ab. ETAR-Ab: anti-Endothelin receptor-Ab. α1R-Ab: anti-α-Adrenergic receptors Ab. α2R-Ab: anti-α2-Adrenergic receptors Ab. β1R-Ab: anti-β1-Adrenergic receptor Ab. β2R-Ab: anti-β2 adrenergic receptor Ab. M1R-Ab: anti Muscarinic Cholinergic receptors-1 Ab. M2R-Ab: anti-Muscarinic Cholinergic Receptors-2 Ab. M3R-Ab: anti-Muscarinic Cholinergic Receptors-3 Ab. M4R-Ab: anti Muscarinic Cholinergic receptors-4 Ab. M5R-Ab anti-Muscarinic Cholinergic receptors-5 Ab. Data were shown as mean values (standard deviation). Units are arbitrary units decided by the kit manufacturer and reported as is from test readout. We report data points, mean mean ±- SD; *N*(LC) = 11, N(APC) = 4.

**Figure 2:**
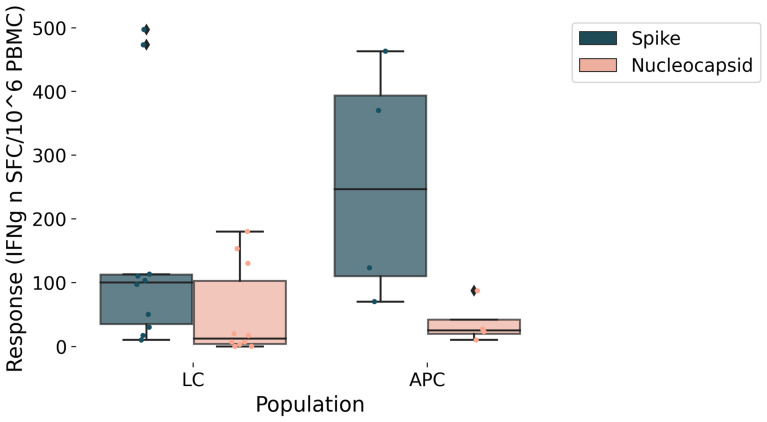
SARS-CoV-2-specific T cell response to Spike and Nucleocapsid proteins in Long COVID and asymptomatic post-COVID patients. The Elispot assay assessed the SARS-CoV-2-specific T-cell response against the Spike and Nucleocapsid proteins. PBMC were stimulated with S and N peptides (1 µg/mL) for 20 h. Data were shown as median values. APC: Asymptomatic Post-COVID, LC: LongCOVID. We report data points as well as 5th, 25th, 50th, 75th, and 95th percentile ranges. *N*(LC) = 11, *N*(APC) = 4.

**Figure 3:**
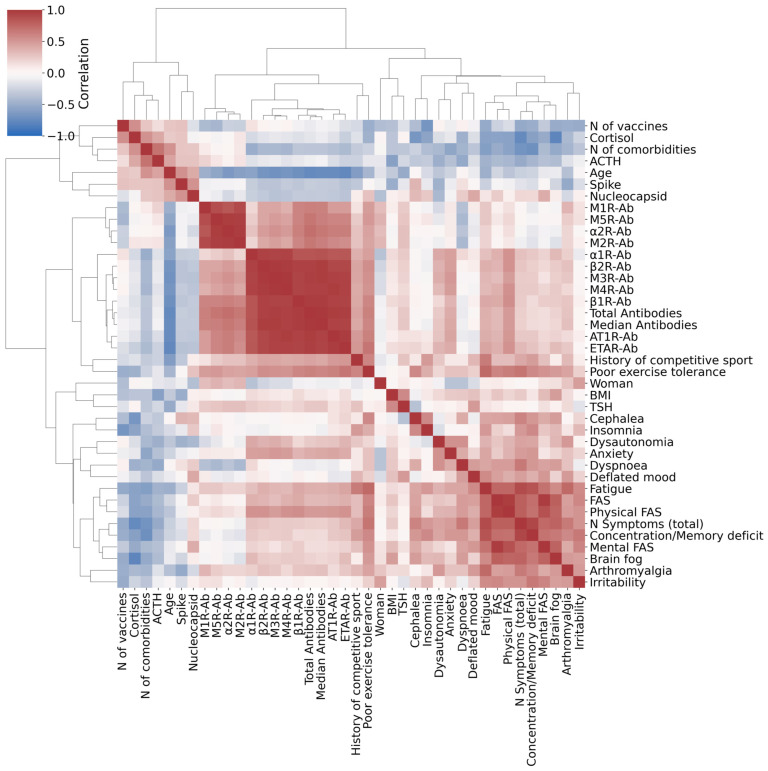
Correlation matrix of clinical, immunological, and endocrine parameters in Long COVID and asymptomatic post-COVID patients. Clustered heatmap showing pairwise Pearson correlations among GPCR autoantibody levels, FAS score, cytokine concentrations, hormonal parameters, and demographic variables. Positive correlations are represented in red and negative correlations in blue, with color intensity proportional to the correlation coefficient. Hierarchical clustering reveals that GPCR autoantibodies (β1-, β2-, α1-, α2-, M1–M4-, AT1R-, and ETAR-Abs) cluster together with fatigue-related symptoms and dysautonomic manifestations, supporting the presence of a shared and biologically meaningful autoimmune signature underlying Long COVID. Cortisol, age, and vaccine dose number inversely correlated with GPCR autoantibody levels and defined the APC group. Sample size of this pilot study was 15 participants. Correlation coefficients are reported in Supplementary Table S1.

Clinical and demographic characteristics of the study population are shown in Table 1. Female sex was the most prevalent for both groups. The mean age of LC patients was 46 years (SD 10), while for APC it was 61 yrs (SD 17) (*p* = 0.2). LC presented fewer comorbidities (*p* = 0.03), had received fewer vaccine doses (*p* = 0.03), and were more likely to engage in sports (*p* = 0.02) before SARS-CoV-2 infection than APC. Mean FAS score was significantly higher in LC than APC (33 vs. 12; *p* = 0.005) as well as both the Physical (17.5 vs. 7; *p* = 0.005) and the Mental (15.7 vs. 5.2; *p* = 0.005) parts of the FAS score. The mean levels of the median value of the studied GPCR AAbs exhibited a trend toward being higher in LC patients compared to APC individuals (8.43 U/mL vs. 5.45 U/mL; *p* = 0.28). This trend was consistent across each specific antibody studied, and anti-β2 adrenergic receptor Ab was the one that differed the most between case and control (Figure 1, Table 2). Morning blood cortisol was statistically higher in APC than LC (17.7 µg/dL (SD 2) vs. 13 µg/dL (SD 3); *p* = 0.01). A trend toward a better specific T cell response to the S and N antigens in APC compared to LC was observed (Figure 2). Correlation analysis revealed a distinct cluster of intercorrelated AAbs (α1R-Ab, β1R-Ab, β2R-Ab, M2R-Ab, M3R-Ab, M4R-Ab, ETAR-Ab, AT1R-Ab), forming an autoimmune pattern that was positively associated with certain LC symptoms (palpitations, orthostatic hypotension, brain fog, poor exercise tolerance, fatigue) and FAS score. In contrast, age and hormonal variables correlated negatively with both immune and symptom clusters, suggesting regulatory influences on the autoimmune–fatigue relationship (Figure 3). We note that, even with the small cohort of APC samples in this study, it is possible to identify 5 out of 10 LC patients based on their GPCR-targeting autoantibodies (Table 2).

We note that, even with the small cohort of APC samples in this study, it is possible to identify 5 out of 10 LC patients based on their GPCR-targeting autoantibodies (Table 2).

## 3. Discussion

Correlation analysis revealed a coherent autoimmune signature characterized by strong intercorrelations among multiple GPCR-targeting and endothelial-directed autoantibodies. This pattern suggests a coordinated autoimmune response rather than independent antibody production, indicating that the concurrent presence of multiple autoantibodies is associated with a greater symptom burden. Notably, this autoimmune cluster showed positive associations with patient-reported fatigue, as measured by the Fatigue Assessment Scale (FAS), as well as with several related functional domains, including dysautonomic manifestations, cognitive impairment (brain fog and concentration/memory deficits), anxiety, and reduced exercise tolerance. Overall, this exploratory study conducted on a subset of participants after SARS-CoV-2 infection suggests a possible association between markers of autoimmune activity and greater subjective fatigue as well as a wider range of Long COVID symptoms. These findings are consistent with the hypothesis that receptor-targeting autoantibodies may be involved in the dysregulation of physiological systems related to energy balance and homeostasis, although further studies are required to confirm this relationship and clarify underlying mechanisms. Accordingly, a recent preprint reported elevated levels of AAbs in the central nervous system of individuals with LC compared with convalescent and healthy controls [9]. However, the specific AAbs involved and the underlying mechanisms of tissue damage have not yet been fully elucidated. Notably, these AAbs showed reactivity against multiple mouse brain structures, including the spinal cord, thalamus, hypothalamus, hindbrain, cerebral nuclei, cerebral cortex, hippocampus, anterior olfactory nucleus, and midbrain, and were associated with neurocognitive and neurological symptoms in patients with LC [9].

Moreover, passive transfer of IgG from patients to mice resulted in pain hypersensitivity and motor impairment resembling clinical features reported in affected individuals [14].

Regulatory AAbs targeting GPCRs have been increasingly recognized as functional modulators of receptor signaling rather than merely epiphenomena of autoimmunity. By acting as agonistic, antagonistic, or allosteric ligands, these autoantibodies can interfere with GPCR-mediated pathways that regulate autonomic, immune, and metabolic functions. Such functional dysregulation may contribute to immune imbalance, autonomic dysfunction, and fluctuating multisystem clinical manifestations observed in autoimmune and post-infectious conditions, including ME/CFS and Long COVID, even in the absence of overt structural organ damage [15]. Among the autoantibodies analyzed, the anti-β_2_R antibody showed the largest difference between LC patients and APC individuals [mean value, respectively: 5.9 U/mL (SD 2.7) vs. 12.9 U/mL (SD 10.4); *p* = 0.18]. Although this difference did not reach statistical significance, it suggests a potential association that warrants further investigation in larger cohorts. The biological relevance of β_2_-ARs supports the plausibility of this observation. β_2_ARs are widely expressed on both innate and adaptive immune cells, including monocytes, macrophages, and lymphocytes [16,17], where they exert predominantly immunomodulatory and anti-inflammatory effects. Under physiological conditions, β_2_-AR activation limits pro-inflammatory cytokine production and contributes to the resolution of immune responses during and after infection [18]. More specifically, β_2_AR signaling appears to differentially regulate immune compartments by enhancing cell-mediated adaptive immune response while downregulating excessive innate immune activation [18]. Experimental studies have shown that β_2_-AR stimulation inhibits lipopolysaccharide-induced TNFα and enhances IL-10 production in human monocytes, thereby protecting animals from lethal endotoxemia [16,19].

The presence of circulating autoantibodies targeting adrenergic receptors may interfere with this regulatory pathway, potentially leading to impaired adrenergic signaling and a reduced ability of monocytes to restrain pro-inflammatory responses [16]. In turn, dysregulated monocyte function may contribute to insufficient control of T-cell activation and proliferation during infection, promoting persistent immune activation or immune imbalance.

Given the widespread expression of β_2_-ARs across immune, endothelial, and autonomic nervous system cells, antibody-mediated β_2_-AR dysfunction could represent a convergent mechanism underlying several hallmark clinical features observed in ME/CFS, including fatigue, dysautonomia, and impaired vascular regulation [18,20]. By extension, similar immune-mediated mechanisms may also contribute to the multisystem manifestations of LC, supporting the hypothesis of partially shared pathophysiological pathways between these conditions [21,22]. Experimental models further support this framework, demonstrating that physiological IgG can modulateβ_2_-AR activity by upregulating receptor activation, inducing anti-inflammatory responses, and exerting a co-stimulatory effect on CD3/CD28-stimulated T cell proliferation [18]. In contrast, patients with ME/CFS exhibiting high circulating levels of AAbs showed little or no modulation of monocyte cytokine production or T cell proliferation, suggesting a loss of normal regulatory function [18].

Consistent with this model, patients with ME/CFS, and similarly those with LC, often experience severe or prolonged infectious episodes [23]. Beyond immune regulation, β_2_-ARs play a key role in vascular tone and the regulation of muscle blood flow during physical exertion [10]. Impaired β_2_-AR signaling in vascular endothelial cells may, therefore, result in paradoxical vasoconstriction upon epinephrine release, due to the unopposed activity of α-adrenergic receptor-mediated vasoconstriction [18].

In this context, immunomodulatory approaches (e.g., immunoabsorption, endovenous immunoglobulin and mTOR inhibitors such as rapamycin) have shown preliminary clinical benefits in selected LC patients with elevated anti-β_2_-AR antibody levels [11,24,25]. However, these observations remain preliminary and require confirmation in controlled studies specifically designed to assess causality and therapeutic efficacy. Conversely, our data revealed negative correlations between endocrine and demographic parameters (vaccination status, blood cortisol levels, and age) and autoimmune or fatigue-related measures, which may reflect impaired HPA-axis regulation in LC patients [13] and age-related immune senescence limiting excessive immune activation [26]. LC predominantly affects young adults, with a higher prevalence among females, who are known to have greater susceptibility to autoimmune diseases, indeed [27]. In line with published evidence, our results support the protective effect of vaccination against LC, attributable to enhanced immunity to SARS-CoV-2 and a decreased incidence of post-infectious complications [28]. In addition, our study showed that the T-cell response to SARS-CoV-2 antigens, measured after stimulation with the Spike (S) and Nucleocapsid (N) proteins, was weaker in patients with LC than in APC individuals. This difference was particularly evident for the response against the Spike protein, which mediates viral entry into host cells. This is in line with a recent study that reported diminished IFN-γ production by CD8^+^ T cells in LC patients after stimulation with the SARS-CoV-2 Spike antigen [29]. These findings may indicate a reduced ability of LC patients to mount an effective antiviral response, leading to impaired viral clearance and prolonged persistence of viral antigens or proteins in the body.

In addition, we explored population-based cut-off values for AAbs, which in this limited cohort were able to identify 5 of 11 patients (45%) with Long COVID without apparent false-positive findings. However, larger studies are required to assess the robustness and generalizability of an AAbs-based scoring approach, including expansion of the study population and incorporation of healthy control groups. Taken together, these preliminary observations support further investigation in this area. Our study has several limitations that should be acknowledged. First, the assessment of autoantibodies was based on binding immunoassays, which do not provide direct information on the functional activity of the antibodies or their agonistic or antagonistic effects on receptor signaling. As such, the observed associations cannot conclusively establish a causal link between GPCR-targeting autoantibodies and the reported clinical manifestations. Future studies incorporating functional assays and receptor signaling analyses are warranted. Second, although the parent cohort comprised 104 individuals, only a limited subset of participants was included in the present sub-study. This selection was driven by feasibility and resource constraints and resulted in a relatively small sample size, which may limit statistical power and the generalizability of the findings. Finally, the cross-sectional and exploratory nature of the study precludes any inference on temporal relationships or disease progression, underscoring the need for larger, longitudinal investigations to validate these findings. Furthermore, differences in age distribution between cases and controls could represent a potential source of bias. Finally, this study was conducted in a selected subgroup of patients with severe LC who exhibited at least five persistent or newly developed symptoms, including fatigue, cognitive impairment, reduced exercise tolerance, dyspnea, arthralgia, or dysautonomic manifestations. Accordingly, the results should be interpreted within this specific clinical context and may not be generalizable to the broader population of individuals with LC. Nevertheless, patients were selected with high accuracy, and the LC cohort was carefully characterized, thereby reducing potential bias from overlap with other comorbidities.

Overall, these results suggest the presence of an immune–LC association in our cohort in which GPCR-targeting autoantibodies tend to cluster and correlate with symptom-intensity scores. This pattern may indicate a potential autoimmune contribution to LC symptoms, which requires further investigation to clarify underlying mechanisms and possible therapeutic implications. Among the autoantibodies analyzed, the anti-β_2_ adrenergic receptor antibody showed the largest difference between LC and asymptomatic individuals; however, this finding should be interpreted with caution and considered hypothesis-generating rather than definitive. Although studies in larger cohorts including healthy control groups are needed, our findings provide preliminary evidence that autoantibodies targeting GPCRs could be explored as candidate markers in future diagnostic approaches for LC. Taken together, these observations are consistent with the involvement of immune-autonomic dysregulation in LC and highlight the need for further mechanistic and interventional studies.

## 4. Materials and Methods

### 4.1. Design of the Study

**Figure 4:**
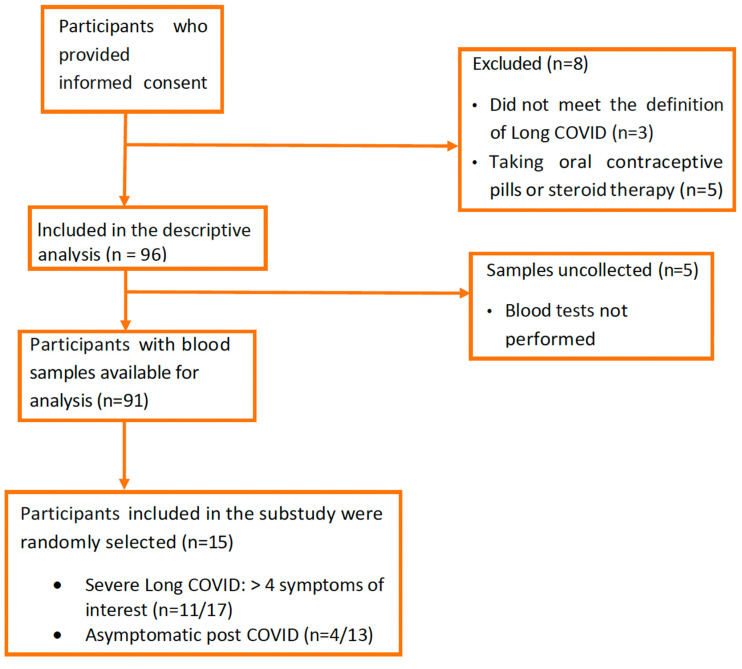
STROBE flow diagram of the study participants (STROBE: Strengthening the Reporting of Observational Studies in Epidemiology).

This is a prospective, single-center, case-control study conducted at the LC clinic of the National Institute for Infectious Diseases L. Spallanzani in Rome, Italy. Between February 2023 and March 2024, 104 consecutive patients were enrolled, referred from home, hospital wards, or the early-treatment SARS-CoV-2 clinic [30]. All included patients were over 18 years old, had a documented previous acute SARS-CoV-2 infection confirmed by a positive swab result at least 28 days before the baseline visit, and had provided signed informed consent. Exclusion criteria were ongoing steroid (oral, injectable, or inhalable) or contraceptive use, and a history of uncontrolled rheumatologic or endocrine disease. We present a sub-analysis of the larger cohort study performed on 15 participants (Figure 4) [13]. Long COVID was defined according to the ECCMID 2022 definition as one or more symptoms and/or signs persisting or relapsing/remitting from 4 to 12 weeks after an acute COVID-19 diagnosis, without an alternative explanation [31].

The cases group was composed of 11 patients diagnosed with severe Long COVID (LC). The control group consisted of four asymptomatic post-COVID (APC) participants.

Asymptomatic post-COVID (APC) individuals were selected as the comparator group to control for prior SARS-CoV-2 infection and to distinguish immunological features associated with persistent symptoms from those related to viral exposure alone.

The primary endpoint of the study was to compare blood levels of GPCR auto-Abs between cases and control groups. The secondary endpoints were to measure differences in SARS-CoV-2-specific T-cell responses, blood morning cortisol levels, and adrenocorticotropic hormone (ACTH) between the study populations.

### 4.2. Procedures

At baseline (BL), a clinical assessment focusing on the burden of Long COVID–related symptoms, daily functional status, and fatigue was performed by an infectious disease specialist, the latter evaluated using the Fatigue Assessment Scale (FAS) [32]. Moreover, comprehensive medical histories were obtained, including details of the acute COVID-19 phase and relevant diagnostic findings. Severe LC was defined by the presence of at least five persistent or newly developed symptoms, such as fatigue, cognitive impairment, poor exercise tolerance, dyspnea, arthralgia, or dysautonomic manifestations, that significantly impaired daily activities. The Fatigue Assessment Scale (FAS) questionnaire was used to assess fatigue, with scores ≥ 22 indicating fatigue and ≥35 indicating severe fatigue.

Two weeks post-BL, early morning fasting blood samples (7:30–8:30 a.m.) were collected for hormonal cortisol and adrenocorticotropic hormone (ACTH) to assess hypothalamic–pituitary–adrenal (HPA) axis function. Hormone levels were measured using a chemiluminescent immunoassay analyzer (CLIA).

Among patients with severe LC (N = 17), 11 participants were randomly selected for the present sub-study. Among APC patients (N = 13), 4 were randomly selected, based on available resources. A higher case-to-control ratio was intentionally maintained to allow participants with LC symptoms and high-titer autoantibodies to potentially benefit over time from off-label immunomodulatory therapy.

So, at the same time point, SARS-CoV-2-specific T-cell responses were evaluated in this subset of the study participants (N = 15) using an Elispot assay, measuring interferon-γ release in response to stimulation with Spike and Nucleocapsid antigens. Briefly, PBMC from LC and APC patients were stimulated with a cocktail of Spike and Nucleacapsid peptides (1 µg/mL, Miltenyi Biotech, Bologna, Italy) for 20 h at 37 °C and 5% of CO_2_. Anti-CD28 and anti-CD49d (1 µg/mL, BD Biosciences, Franklin Lakes, New Jersey, USA) co-stimuli were added to the culture. A Superantigen SEB (200 ng/mL, Staphylococcal Enterotoxin B; Sigma-Merck, Milano, Italy) was included as a positive control. At the end of incubation, the ELISpot assay was developed according to the manufacturer’s instructions (Human IFN-γ ELISpot plus kit; Mabtech, Nacka Strand, Sweden). Spontaneous cytokine production (background) was assessed by incubating PBMCs with DMSO, the peptides’ diluent (Sigma-Merck, Milano, Italy). Results are expressed as spot-forming cells (SFC) per 10^6^ PBMCs in stimulating cultures after subtracting the background.

Serum sample of the following autoantibodies were tested for each patient between January and March 2024: anti Angiotensin II type 1 receptor (AT1R)-Ab, anti-Endothelin receptor A (ETAR)-Ab, anti α1 Adrenergic receptors Ab, anti α2 Adrenergic receptors Ab, anti β1 Adrenergic receptors Ab, anti β2 Adrenergic receptors Ab, anti-Muscarinic Cholinergic receptors-1 (M1) Ab, anti-Muscarinic Cholinergic receptors-2 (M2) Ab, anti-Muscarinic Cholinergic receptors-3 (M3) Ab, anti-Muscarinic Cholinergic receptors-4 (M4) Ab, anti-Muscarinic Cholinergic receptors-5 (M5) Ab.

Autoantibodies were measured using ELISA technology (CellTrend GmbH, Luckenwalde, Germany). Upper normal limits were established based on validation studies in a healthy control group and defined as values exceeding the 90th percentile.

### 4.3. Statistical Analysis

For the descriptive demographic tables, *p*-values were calculated using the Mann–Whitney U test. The values were not adjusted for false discovery rate (FDR) or family-wise error rate (FWER), as this is only a descriptive and not actionable comparison. Antibody levels are reported with the kit manufacturer’s specified units to allow further usage in clinical settings. To investigate the covariances across our data, we studied the set of pairwise Pearson correlation coefficients. We plotted them in a clustered heatmap (clustermap) using the Python seaborn (version 0.12.2) package [33]. The clusters were assigned via hierarchical clustering with Euclidean distance metrics and the ‘complete’ method (for details, see scipy (version 1.15.2) clustering [34]). The cluster map summarizes all linear relationships among all variables studied and allows for a visual representation of inferred coherent behavior across variables. To develop our LC detection method based on GPCR-Ab levels, we first constructed a Student’s T distribution for each antibody from the APC patient’s data. Then we set a cutoff at the 95th percentile of this distribution (often higher than the accepted physiological range). The choice of the 95th percentile is based on the scientific consensus of considering *p*-values of 0.05 significant. Therefore, this cutoff that is one-tailed has been set at the 95th percentile of the inferred Student’s T distribution a priori; we then verified that such a cutoff works well for this dataset and needed no further tuning. If any of the GPCR-Ab levels fell above the defined cutoffs, we can label the sample as LC. This is a strict measurement with no false positives and a 45% success rate in identifying the presence of LC in our dataset. Although there is no statistical difference among the means of APC and LC for any antibody, this does not mean or imply that no information can be extracted from antibodies data. In fact, if two distributions have the same mean but different standard deviations, it is possible to use parameters from the tighter distributions to reliably infer outliers belonging to the wider distributions. This concept is at the base of our LC diagnosis based on antibody measurements.

## Figures and Tables

**Table 1:** Demographic and clinical metadata of patients enrolled in the study. * n (%); ** mean (standard deviation). APC: Asymptomatic post-COVID. LC: LongCOVID. BMI: Body Mass Index. FAS: Fatigue Assessment Scale. AAbs: autoantibodies. Blood cortisol (µg/dL). ACTH: adrenocorticotropic hormone (pg/mL). *N* (LC) = 11, N(APC) = 4. Variables analysed and statistically significant *p* values are shown in bold.

Variables	APC	LC	*p*-Value
Participants *	4 (28%)	11 (73%)	1
Female sex *	3 (75%)	9 (82%)	1
Age **	61 (17)	49 (15)	0.15
BMI **	23.6 (2.6)	24.5 (2.4)	0.7
History of competitive sports *	0	8 (73%)	**0.02**
N of comorbidities **	4 (1.2)	2 (1.1)	**0.03**
N of vaccine doses **	3.7 (0.4)	2.6 (0.8)	**0.03**
FAS score **	12 (2.1)	33 (9.3)	**0.005**
Mental FAS **	5.2 (0.4)	15.7 (4.6)	**0.005**
Physical FAS **	7 (1.7)	17.5 (4.6)	**0.005**
Total AAbs **	5.4 (1.9)	8.8 (4.2)	0.21
Cortisol 8:00 a.m. (µg/dL) **	17.7 (2)	13 (3)	**0.01**
ACTH (pg/mL) **	22 (5.9)	14 (6.9)	0.11

**Table 2:** Autoantibodies distribution in the study population and diagnostic cut-offs. Mean value (standard deviation) for the set of measured antibodies in the two populations (APC, LC). AAbs = Autoantibodies. APC: Asymptomatic Post-COVID. LC: LongCOVID. AT1R-Ab: anti-Angiotensin II type 1 receptor-Ab. ETAR-Ab: anti-Endothelin receptor-Ab. α1R-Ab: anti-α-Adrenergic receptors Ab. α2R-Ab: anti-α2-Adrenergic receptors Ab. β1R-Ab: anti-β1-Adrenergic receptor Ab. β2R-Ab: anti-β2 adrenergic receptor Ab. M1R-Ab: anti Muscarinic Cholinergic receptors-1 Ab. M2R-Ab: anti-Muscarinic Cholinergic Receptors-2 Ab. M3R-Ab: anti-Muscarinic Cholinergic Receptors-3 Ab. M4R-Ab: anti Muscarinic Cholinergic receptors-4 A. M5R-Ab anti Muscarinic Cholinergic receptors-5 Ab. *p*-values were calculated between the two distributions using the non-parametric Mann Whitney u test. The LC diagnostic cut-off was calculated taking the 95th percentile of the inferred Student’s T distribution of the APC samples. One AAb value over the cutoff is required for an LC diagnosis. Studied variables and diagnostic cut-off values are shown in bold.

AAbs Variables	APC	LC	*p*-Value	Diagnostic Cut-Off
**AT1R-Ab**	8.45 (1.6)	11.48 (3.6)	0.30	**12.27**
**ETAR-Ab**	8.55 (1.9)	12.3 (3.9)	0.13	**12.95**
**α1R-Ab**	4.42 (1.3)	7.99 (5.7)	0.17	**7.92**
**α2R-Ab**	7.12 (3.7)	8.71 (3.3)	0.41	**16.27**
**β1R-Ab**	5.05 (1.7)	10 (5.7)	0.17	**9.65**
**β2R-Ab**	5.92 (2.3)	13.6 (9.6)	0.13	**12.30**
**M1R-Ab**	2.37 (1)	3.58 (2.1)	0.29	**5.18**
**M2R-Ab**	5.07 (2.2)	6.3 (2.6)	0.41	**10.97**
**M3R-Ab**	4.52 (1.1)	6.86 (3.3)	0.32	**7.47**
**M4R-Ab**	5.02 (1.9)	8.67 (5.2)	0.22	**10.16**
**M5R-Ab**	7.2 (3.5)	9.72 (4.3)	0.28	**16.80**
**Total AAbs**	63.72 (20.3)	99.29 (42.6)	0.17	**118.98**
**Median AAbs**	5.45 (1.9)	8.88 (4.2)	0.17	**10.75**

## Data Availability

Clinical, epidemiological, demographic and laboratory data were anonymized and recorded using electronic case report forms (eCRFs) (Horfavid Study). Data are available upon reasonable request.

## Supplementary Materials

The following supporting information can be downloaded at: https://www.mdpi.com/article/10.3390/ijms27041787/s1.
