# Supplementary material for: Prevalence of Circulating Autoantibodies Against G-Protein-Coupled Receptors as Potential Biomarkers for Long COVID: Preliminary Investigations
Source: Int J Mol Sci. 2026 Feb 13;27(4):1787. doi: 10.3390/ijms27041787 (PMC12940979; doi:10.3390/ijms27041787)
Supplement: Supplementary file 1 [file ijms-27-01787-s001.zip › ijms-4072484-supplementary.pdf]

Supplements

Prevalence of circulating autoantibodies targeting G-protein-coupled neurotransmitter receptors: can it be a reliable biomarker for Long COVID patients?

Marta Camici[1]. Marta Franco[2]. Lorenzo Talamanca[3]. Jessica Paulicelli[1]. Liliana Scarnecchia[4]. Manuela Petino[8]. Valentina Mazzotta[1]. Ilaria Mastrorosa[1]. Eleonora Cimini*[5]. Eleonora Tartaglia[5]. Stefania Notari[5]. Paolo Zuppi[9]. Roberto Baldelli[4]. Maria Grazia Bocci[10]. Fabrizio Maggi[6]. Enrico Girardi[7]. Andrea Antinori[1]

Supplementary Table S1. Correlation coefficients of clinical, immunological, and endocrine parameters in Long COVID and asymptomatic post-COVID participants.

Element 1	Element 2	Correlation	P-value
BMI	N of comorbidities	-0.33597	0.261727
BMI	N of vaccines	-0.37078	0.212313
BMI	History of competitive sport	-0.05539	0.857375
BMI	Woman	-0.01503	0.96112
BMI	Age	-0.30599	0.309264
BMI	FAS	0.266411	0.378951
BMI	Mental FAS	0.315787	0.293228
BMI	Physical FAS	0.192789	0.528017
BMI	AT1R-Ab	0.064902	0.83316
BMI	ETAR-Ab	0.152497	0.618936
BMI	α1R-Ab	0.163812	0.592818
BMI	α2R-Ab	-0.04982	0.871597
BMI	β1R-Ab	0.165075	0.58993
BMI	β2R-Ab	0.129047	0.674362
BMI	M1R-Ab	0.073565	0.811232
BMI	M2R-Ab	-0.04617	0.880933
BMI	M3R-Ab	0.097984	0.750138
BMI	M4R-Ab	0.092541	0.763654
BMI	M5R-Ab	0.015087	0.960984
BMI	Total Antibodies	0.105999	0.730358
BMI	Median Antibodies	0.105401	0.73183
BMI	N Symptoms (total)	0.242757	0.424208
BMI	Dyspnoea	0.256301	0.397973
BMI	Cephalea	-0.07946	0.796378
BMI	Concentration/Memory deficit	0.115521	0.707059
BMI	Dysautonomia	0.17302	0.571893
BMI	Fatigue	0.160762	0.599816
BMI	Insomnia	0.096296	0.754324
BMI	Anxiety	0.249088	0.411839
BMI	Deflated mood	0.293097	0.331117
BMI	Arthromyalgia	0.160762	0.599816
BMI	Poor exercise tolerance	0.021389	0.944706
BMI	Irritability	0.009574	0.975237
BMI	Brain fog	0.470374	0.104778
BMI	Spike	-0.50673	0.077189
BMI	Nucleocapside	0.058731	0.848853
BMI	Cortisol	-0.33866	0.257689
BMI	ACTH	-0.59804	0.039977
BMI	TSH	0.572803	0.040745
N of comorbidities	N of vaccines	0.273133	0.324641
N of comorbidities	History of competitive sport	-0.30012	0.277111
N of comorbidities	Woman	-0.04583	0.871147
N of comorbidities	Age	0.528954	0.042619
N of comorbidities	FAS	-0.51042	0.051883
N of comorbidities	Mental FAS	-0.41177	0.127248

N of comorbidities	Physical FAS	-0.58061	0.023241
N of comorbidities	AT1R-Ab	-0.37075	0.173702
N of comorbidities	ETAR-Ab	-0.41006	0.128998
N of comorbidities	α1R-Ab	-0.50697	0.053757
N of comorbidities	α2R-Ab	0.018383	0.948154
N of comorbidities	β1R-Ab	-0.33518	0.221993
N of comorbidities	β2R-Ab	-0.41727	0.121749
N of comorbidities	M1R-Ab	0.091328	0.746163
N of comorbidities	M2R-Ab	0.109131	0.698642
N of comorbidities	M3R-Ab	-0.42395	0.115285
N of comorbidities	M4R-Ab	-0.44385	0.09745
N of comorbidities	M5R-Ab	0.027543	0.92238
N of comorbidities	Total Antibodies	-0.33762	0.218442
N of comorbidities	Median Antibodies	-0.34169	0.212589
N of comorbidities	N Symptoms (total)	-0.69428	0.004082
N of comorbidities	Dyspnoea	-0.45374	0.08935
N of comorbidities	Cephalea	-0.25928	0.350731
N of comorbidities	Concentration/Memory deficit	-0.71302	0.002845
N of comorbidities	Dysautonomia	-0.42841	0.111107
N of comorbidities	Fatigue	-0.60807	0.016174
N of comorbidities	Insomnia	-0.39295	0.14736
N of comorbidities	Anxiety	-0.55097	0.033278
N of comorbidities	Deflated mood	-0.343	0.210725
N of comorbidities	Arthromyalgia	-0.44909	0.0931
N of comorbidities	Poor exercise tolerance	-0.26197	0.345581
N of comorbidities	Irritability	-0.343	0.210725
N of comorbidities	Brain fog	-0.52675	0.043653
N of comorbidities	Spike	0.270624	0.349374
N of comorbidities	Nucleocapside	0.2581	0.372973
N of comorbidities	Cortisol	0.533589	0.040509
N of comorbidities	ACTH	0.694909	0.005806
N of comorbidities	TSH	-0.33032	0.22918
N of vaccines	History of competitive sport	-0.21107	0.450175
N of vaccines	Woman	-0.39488	0.145211
N of vaccines	Age	0.269762	0.330886
N of vaccines	FAS	-0.26357	0.342531
N of vaccines	Mental FAS	-0.31115	0.258971
N of vaccines	Physical FAS	-0.19636	0.48305
N of vaccines	AT1R-Ab	-0.10016	0.722481
N of vaccines	ETAR-Ab	-0.18218	0.515799
N of vaccines	α1R-Ab	0.129877	0.644557
N of vaccines	α2R-Ab	-0.26823	0.333737
N of vaccines	β1R-Ab	-0.075	0.7905
N of vaccines	β2R-Ab	0.037141	0.895451
N of vaccines	M1R-Ab	-0.40711	0.132052
N of vaccines	M2R-Ab	-0.28361	0.305669

N of vaccines	M3R-Ab	-0.01322	0.96269
N of vaccines	M4R-Ab	0.036825	0.896336
N of vaccines	M5R-Ab	-0.44172	0.099267
N of vaccines	Total Antibodies	-0.11322	0.687874
N of vaccines	Median Antibodies	-0.06411	0.820437
N of vaccines	N Symptoms (total)	-0.5308	0.04177
N of vaccines	Dyspnoea	0.050767	0.857406
N of vaccines	Cephalea	-0.40614	0.133062
N of vaccines	Concentration/Memory deficit	-0.50767	0.053373
N of vaccines	Dysautonomia	0.043295	0.878239
N of vaccines	Fatigue	-0.53036	0.041972
N of vaccines	Insomnia	-0.67414	0.005849
N of vaccines	Anxiety	-0.10153	0.718803
N of vaccines	Deflated mood	-0.07675	0.785718
N of vaccines	Arthromyalgia	-0.49828	0.058703
N of vaccines	Poor exercise tolerance	-0.49828	0.058703
N of vaccines	Irritability	-0.50849	0.052927
N of vaccines	Brain fog	-0.50849	0.052927
N of vaccines	Spike	0.285673	0.32214
N of vaccines	Nucleocapside	-0.17938	0.539472
N of vaccines	Cortisol	0.542425	0.036701
N of vaccines	ACTH	0.15363	0.600023
N of vaccines	TSH	-0.16473	0.55743
History of competitive sport	Woman	-0.13363	0.634938
History of competitive sport	Age	-0.61865	0.013945
History of competitive sport	FAS	0.312386	0.256977
History of competitive sport	Mental FAS	0.30013	0.277099
History of competitive sport	Physical FAS	0.304814	0.269301
History of competitive sport	AT1R-Ab	0.491015	0.063083
History of competitive sport	ETAR-Ab	0.506873	0.053812
History of competitive sport	α1R-Ab	0.381041	0.161133
History of competitive sport	α2R-Ab	0.287249	0.299232
History of competitive sport	β1R-Ab	0.416347	0.122662
History of competitive sport	β2R-Ab	0.434806	0.105301
History of competitive sport	M1R-Ab	0.127171	0.651525
History of competitive sport	M2R-Ab	0.282013	0.308517
History of competitive sport	M3R-Ab	0.42549	0.113834
History of competitive sport	M4R-Ab	0.403018	0.136356
History of competitive sport	M5R-Ab	0.245687	0.377434
History of competitive sport	Total Antibodies	0.439534	0.101145
History of competitive sport	Median Antibodies	0.415986	0.12302
History of competitive sport	N Symptoms (total)	0.407145	0.132011
History of competitive sport	Dyspnoea	0.094491	0.737652
History of competitive sport	Cephalea	0.094491	0.737652
History of competitive sport	Concentration/Memory deficit	0.472456	0.075346
History of competitive sport	Dysautonomia	0.261892	0.345726

History of competitive sport	Fatigue	0.644658	0.009472
History of competitive sport	Insomnia	0.49099	0.063099
History of competitive sport	Anxiety	0.094491	0.737652
History of competitive sport	Deflated mood	0.339286	0.216029
History of competitive sport	Arthromyalgia	0.327327	0.233688
History of competitive sport	Poor exercise tolerance	0.600099	0.018026
History of competitive sport	Irritability	0.071429	0.800296
History of competitive sport	Brain fog	0.071429	0.800296
History of competitive sport	Spike	-0.32338	0.259394
History of competitive sport	Nucleocapside	0.073418	0.80303
History of competitive sport	Cortisol	-0.17083	0.542716
History of competitive sport	ACTH	-0.25812	0.372927
History of competitive sport	TSH	0.280583	0.311081
Woman	Age	-0.15656	0.577397
Woman	FAS	0.089267	0.751723
Woman	Mental FAS	0.126533	0.653172
Woman	Physical FAS	0.044292	0.875454
Woman	AT1R-Ab	-0.09538	0.735274
Woman	ETAR-Ab	-0.06104	0.828916
Woman	α1R-Ab	-0.36665	0.178876
Woman	α2R-Ab	0.310187	0.26052
Woman	β1R-Ab	-0.08399	0.766007
Woman	β2R-Ab	-0.24789	0.373027
Woman	M1R-Ab	0.312264	0.257173
Woman	M2R-Ab	0.337559	0.218524
Woman	M3R-Ab	-0.19626	0.483274
Woman	M4R-Ab	-0.20174	0.470889
Woman	M5R-Ab	0.360394	0.186974
Woman	Total Antibodies	-0.06441	0.819594
Woman	Median Antibodies	-0.09421	0.738413
Woman	N Symptoms (total)	-0.01444	0.959265
Woman	Dyspnoea	-0.35355	0.196096
Woman	Cephalea	1.39E-17	1
Woman	Concentration/Memory deficit	3.47E-17	1
Woman	Dysautonomia	-0.07538	0.789477
Woman	Fatigue	0.075378	0.789477
Woman	Insomnia	0.068041	0.809602
Woman	Anxiety	-0.35355	0.196096
Woman	Deflated mood	-0.20045	0.473809
Woman	Arthromyalgia	0.272166	0.326426
Woman	Poor exercise tolerance	-0.06804	0.809602
Woman	Irritability	0.467707	0.078739
Woman	Brain fog	0.133631	0.634938
Woman	Spike	-0.09453	0.747878
Woman	Nucleocapside	-0.38011	0.180038
Woman	Cortisol	0.031179	0.912167

Woman	ACTH	-0.13743	0.639434
Woman	TSH	0.086403	0.759469
Age	FAS	-0.23546	0.398229
Age	Mental FAS	-0.12169	0.665728
Age	Physical FAS	-0.33952	0.215686
Age	AT1R-Ab	-0.73819	0.001676
Age	ETAR-Ab	-0.77239	0.000738
Age	α1R-Ab	-0.57636	0.024518
Age	α2R-Ab	-0.64146	0.009951
Age	β1R-Ab	-0.71336	0.002826
Age	β2R-Ab	-0.68423	0.004901
Age	M1R-Ab	-0.48532	0.066679
Age	M2R-Ab	-0.59526	0.019227
Age	M3R-Ab	-0.698	0.003807
Age	M4R-Ab	-0.67543	0.005721
Age	M5R-Ab	-0.55015	0.033596
Age	Total Antibodies	-0.75429	0.001158
Age	Median Antibodies	-0.74004	0.001608
Age	N Symptoms (total)	-0.27806	0.315639
Age	Dyspnoea	0.028466	0.919786
Age	Cephalea	0.009489	0.973227
Age	Concentration/Memory deficit	-0.2562	0.356698
Age	Dysautonomia	-0.3338	0.224028
Age	Fatigue	-0.48552	0.066551
Age	Insomnia	-0.21	0.452524
Age	Anxiety	-0.2562	0.356698
Age	Deflated mood	-0.11656	0.679109
Age	Arthromyalgia	-0.37435	0.169229
Age	Poor exercise tolerance	-0.43827	0.102249
Age	Irritability	-0.03586	0.899027
Age	Brain fog	-0.11656	0.679109
Age	Spike	0.538576	0.04692
Age	Nucleocapside	0.400309	0.1561
Age	Cortisol	0.248554	0.371714
Age	ACTH	0.443677	0.112032
Age	TSH	-0.51973	0.047064
FAS	Mental FAS	0.969628	2.5E-09
FAS	Physical FAS	0.966453	4.73E-09
FAS	AT1R-Ab	0.34484	0.208123
FAS	ETAR-Ab	0.376545	0.166545
FAS	α1R-Ab	0.37144	0.172835
FAS	α2R-Ab	0.039208	0.889662
FAS	β1R-Ab	0.398718	0.140987
FAS	β2R-Ab	0.358582	0.189363
FAS	M1R-Ab	0.059124	0.834213
FAS	M2R-Ab	0.023807	0.932884

FAS	M3R-Ab	0.305962	0.26741
FAS	M4R-Ab	0.368921	0.175996
FAS	M5R-Ab	0.107084	0.704056
FAS	Total Antibodies	0.32758	0.233305
FAS	Median Antibodies	0.32656	0.23485
FAS	N Symptoms (total)	0.795473	0.000391
FAS	Dyspnoea	0.477356	0.071955
FAS	Cephalea	0.44185	0.099152
FAS	Concentration/Memory deficit	0.718006	0.002573
FAS	Dysautonomia	0.270833	0.328894
FAS	Fatigue	0.751098	0.001248
FAS	Insomnia	0.218659	0.433663
FAS	Anxiety	0.24065	0.3876
FAS	Deflated mood	0.45926	0.085044
FAS	Arthromyalgia	0.453262	0.089732
FAS	Poor exercise tolerance	0.601312	0.017734
FAS	Irritability	0.582276	0.022755
FAS	Brain fog	0.794758	0.000399
FAS	Spike	-0.04742	0.87211
FAS	Nucleocapside	0.238358	0.411856
FAS	Cortisol	-0.57094	0.02622
FAS	ACTH	-0.41228	0.142954
FAS	TSH	0.089198	0.751909
Mental FAS	Physical FAS	0.87428	2.02E-05
Mental FAS	AT1R-Ab	0.174443	0.534074
Mental FAS	ETAR-Ab	0.206876	0.459435
Mental FAS	α1R-Ab	0.201739	0.4709
Mental FAS	α2R-Ab	-0.06669	0.813333
Mental FAS	β1R-Ab	0.229854	0.409877
Mental FAS	β2R-Ab	0.177706	0.526329
Mental FAS	M1R-Ab	-0.02059	0.941933
Mental FAS	M2R-Ab	-0.0551	0.845359
Mental FAS	M3R-Ab	0.124655	0.658029
Mental FAS	M4R-Ab	0.187562	0.503251
Mental FAS	M5R-Ab	0.028858	0.918684
Mental FAS	Total Antibodies	0.153346	0.585323
Mental FAS	Median Antibodies	0.15056	0.592236
Mental FAS	N Symptoms (total)	0.749573	0.001294
Mental FAS	Dyspnoea	0.447363	0.094519
Mental FAS	Cephalea	0.402626	0.136774
Mental FAS	Concentration/Memory deficit	0.693412	0.004147
Mental FAS	Dysautonomia	0.181218	0.518051
Mental FAS	Fatigue	0.701029	0.003595
Mental FAS	Insomnia	0.266894	0.33625
Mental FAS	Anxiety	0.111841	0.691494
Mental FAS	Deflated mood	0.503034	0.055958

Mental FAS	Arthromyalgia	0.400342	0.139226
Mental FAS	Poor exercise tolerance	0.529484	0.042374
Mental FAS	Irritability	0.608714	0.016031
Mental FAS	Brain fog	0.777801	0.00064
Mental FAS	Spike	-0.01199	0.967541
Mental FAS	Nucleocapside	0.395305	0.161824
Mental FAS	Cortisol	-0.52311	0.045396
Mental FAS	ACTH	-0.36958	0.193414
Mental FAS	TSH	0.133433	0.635442
Physical FAS	AT1R-Ab	0.50121	0.057
Physical FAS	ETAR-Ab	0.530076	0.042101
Physical FAS	α1R-Ab	0.525339	0.044322
Physical FAS	α2R-Ab	0.147844	0.599009
Physical FAS	β1R-Ab	0.549953	0.033674
Physical FAS	β2R-Ab	0.525057	0.044457
Physical FAS	M1R-Ab	0.138965	0.621358
Physical FAS	M2R-Ab	0.105114	0.709281
Physical FAS	M3R-Ab	0.476333	0.072653
Physical FAS	M4R-Ab	0.535227	0.039781
Physical FAS	M5R-Ab	0.182224	0.515693
Physical FAS	Total Antibodies	0.48911	0.064271
Physical FAS	Median Antibodies	0.49001	0.063707
Physical FAS	N Symptoms (total)	0.791616	0.000437
Physical FAS	Dyspnoea	0.477613	0.07178
Physical FAS	Cephalea	0.454124	0.089047
Physical FAS	Concentration/Memory deficit	0.696845	0.003891
Physical FAS	Dysautonomia	0.347215	0.204799
Physical FAS	Fatigue	0.754525	0.001151
Physical FAS	Insomnia	0.153697	0.584454
Physical FAS	Anxiety	0.360167	0.187272
Physical FAS	Deflated mood	0.383237	0.158532
Physical FAS	Arthromyalgia	0.479172	0.070726
Physical FAS	Poor exercise tolerance	0.637389	0.010589
Physical FAS	Irritability	0.516408	0.048743
Physical FAS	Brain fog	0.760555	0.000995
Physical FAS	Spike	-0.08024	0.785096
Physical FAS	Nucleocapside	0.055208	0.851307
Physical FAS	Cortisol	-0.58381	0.022315
Physical FAS	ACTH	-0.43007	0.124805
Physical FAS	TSH	0.036909	0.8961
AT1R-Ab	ETAR-Ab	0.976272	5.1E-10
AT1R-Ab	α1R-Ab	0.829283	0.000132
AT1R-Ab	α2R-Ab	0.633306	0.01126
AT1R-Ab	β1R-Ab	0.938154	2.36E-07
AT1R-Ab	β2R-Ab	0.920784	1.13E-06
AT1R-Ab	M1R-Ab	0.478798	0.070978

AT1R-Ab	M2R-Ab	0.573916	0.025274
AT1R-Ab	M3R-Ab	0.951928	4.74E-08
AT1R-Ab	M4R-Ab	0.961991	1.05E-08
AT1R-Ab	M5R-Ab	0.598294	0.018467
AT1R-Ab	Total Antibodies	0.964357	6.98E-09
AT1R-Ab	Median Antibodies	0.94378	1.28E-07
AT1R-Ab	N Symptoms (total)	0.251433	0.366016
AT1R-Ab	Dyspnoea	-0.11717	0.677504
AT1R-Ab	Cephalea	0.132158	0.638704
AT1R-Ab	Concentration/Memory deficit	0.252055	0.364792
AT1R-Ab	Dysautonomia	0.279149	0.313666
AT1R-Ab	Fatigue	0.387496	0.153566
AT1R-Ab	Insomnia	0.100687	0.721064
AT1R-Ab	Anxiety	0.430537	0.109154
AT1R-Ab	Deflated mood	-0.13955	0.619865
AT1R-Ab	Arthromyalgia	0.158896	0.571647
AT1R-Ab	Poor exercise tolerance	0.6112	0.015488
AT1R-Ab	Irritability	0.065143	0.817585
AT1R-Ab	Brain fog	0.204182	0.465431
AT1R-Ab	Spike	-0.30946	0.281637
AT1R-Ab	Nucleocapside	-0.32409	0.258297
AT1R-Ab	Cortisol	-0.159	0.571385
AT1R-Ab	ACTH	-0.18789	0.520074
AT1R-Ab	TSH	0.131188	0.641193
ETAR-Ab	α1R-Ab	0.842499	8.08E-05
ETAR-Ab	α2R-Ab	0.634463	0.011067
ETAR-Ab	β1R-Ab	0.93015	5.09E-07
ETAR-Ab	β2R-Ab	0.920014	1.2E-06
ETAR-Ab	M1R-Ab	0.480867	0.069594
ETAR-Ab	M2R-Ab	0.552939	0.032526
ETAR-Ab	M3R-Ab	0.942495	1.48E-07
ETAR-Ab	M4R-Ab	0.93925	2.1E-07
ETAR-Ab	M5R-Ab	0.595795	0.019092
ETAR-Ab	Total Antibodies	0.960238	1.41E-08
ETAR-Ab	Median Antibodies	0.936089	2.9E-07
ETAR-Ab	N Symptoms (total)	0.332028	0.226642
ETAR-Ab	Dyspnoea	-0.0148	0.958256
ETAR-Ab	Cephalea	0.188673	0.500678
ETAR-Ab	Concentration/Memory deficit	0.277461	0.316723
ETAR-Ab	Dysautonomia	0.350985	0.199593
ETAR-Ab	Fatigue	0.433802	0.106198
ETAR-Ab	Insomnia	0.131713	0.639844
ETAR-Ab	Anxiety	0.392145	0.148268
ETAR-Ab	Deflated mood	-0.11885	0.673111
ETAR-Ab	Arthromyalgia	0.252747	0.363431
ETAR-Ab	Poor exercise tolerance	0.672807	0.005985

ETAR-Ab	Irritability	0.010487	0.970411
ETAR-Ab	Brain fog	0.318107	0.247899
ETAR-Ab	Spike	-0.36315	0.201879
ETAR-Ab	Nucleocapside	-0.347	0.224168
ETAR-Ab	Cortisol	-0.28363	0.305626
ETAR-Ab	ACTH	-0.26467	0.360484
ETAR-Ab	TSH	0.177605	0.526568
α1R-Ab	α2R-Ab	0.286203	0.301074
α1R-Ab	β1R-Ab	0.84536	7.22E-05
α1R-Ab	β2R-Ab	0.95261	4.32E-08
α1R-Ab	M1R-Ab	0.183848	0.511891
α1R-Ab	M2R-Ab	0.191847	0.493366
α1R-Ab	M3R-Ab	0.936406	2.81E-07
α1R-Ab	M4R-Ab	0.938198	2.34E-07
α1R-Ab	M5R-Ab	0.229853	0.409878
α1R-Ab	Total Antibodies	0.84448	7.48E-05
α1R-Ab	Median Antibodies	0.866576	2.92E-05
α1R-Ab	N Symptoms (total)	0.293256	0.288785
α1R-Ab	Dyspnoea	0.176153	0.530008
α1R-Ab	Cephalea	-0.02981	0.916009
α1R-Ab	Concentration/Memory deficit	0.252035	0.364832
α1R-Ab	Dysautonomia	0.408494	0.130611
α1R-Ab	Fatigue	0.302181	0.273667
α1R-Ab	Insomnia	-0.07667	0.785951
α1R-Ab	Anxiety	0.460709	0.083938
α1R-Ab	Deflated mood	0.100382	0.721878
α1R-Ab	Arthromyalgia	0.220094	0.430575
α1R-Ab	Poor exercise tolerance	0.454791	0.08852
α1R-Ab	Irritability	-0.1096	0.697402
α1R-Ab	Brain fog	0.325728	0.236115
α1R-Ab	Spike	-0.29508	0.305749
α1R-Ab	Nucleocapside	-0.2857	0.322095
α1R-Ab	Cortisol	-0.21505	0.441477
α1R-Ab	ACTH	-0.16555	0.571652
α1R-Ab	TSH	0.248252	0.372314
α2R-Ab	β1R-Ab	0.621204	0.013445
α2R-Ab	β2R-Ab	0.477247	0.072029
α2R-Ab	M1R-Ab	0.842433	8.1E-05
α2R-Ab	M2R-Ab	0.975165	6.85E-10
α2R-Ab	M3R-Ab	0.529416	0.042405
α2R-Ab	M4R-Ab	0.489252	0.064182
α2R-Ab	M5R-Ab	0.962334	9.95E-09
α2R-Ab	Total Antibodies	0.717637	0.002593
α2R-Ab	Median Antibodies	0.666257	0.006687
α2R-Ab	N Symptoms (total)	-0.04157	0.883067
α2R-Ab	Dyspnoea	-0.35066	0.200043

α2R-Ab	Cephalea	0.001397	0.996057
α2R-Ab	Concentration/Memory deficit	-0.13132	0.640849
α2R-Ab	Dysautonomia	-0.01668	0.952953
α2R-Ab	Fatigue	0.208793	0.455192
α2R-Ab	Insomnia	0.001613	0.995448
α2R-Ab	Anxiety	0.081028	0.77406
α2R-Ab	Deflated mood	-0.1328	0.637062
α2R-Ab	Arthromyalgia	0.191966	0.493091
α2R-Ab	Poor exercise tolerance	0.44604	0.095617
α2R-Ab	Irritability	-0.02587	0.927073
α2R-Ab	Brain fog	-0.08924	0.751803
α2R-Ab	Spike	-0.06911	0.814389
α2R-Ab	Nucleocapside	-0.22156	0.446512
α2R-Ab	Cortisol	-0.00171	0.995187
α2R-Ab	ACTH	0.04563	0.876905
α2R-Ab	TSH	0.298359	0.280081
β1R-Ab	β2R-Ab	0.95209	4.63E-08
β1R-Ab	M1R-Ab	0.60193	0.017587
β1R-Ab	M2R-Ab	0.587323	0.02133
β1R-Ab	M3R-Ab	0.932191	4.22E-07
β1R-Ab	M4R-Ab	0.936492	2.79E-07
β1R-Ab	M5R-Ab	0.603561	0.017203
β1R-Ab	Total Antibodies	0.973888	9.45E-10
β1R-Ab	Median Antibodies	0.97441	8.3E-10
β1R-Ab	N Symptoms (total)	0.236473	0.396141
β1R-Ab	Dyspnoea	-0.10924	0.698341
β1R-Ab	Cephalea	0.046819	0.868402
β1R-Ab	Concentration/Memory deficit	0.163867	0.559514
β1R-Ab	Dysautonomia	0.17801	0.525611
β1R-Ab	Fatigue	0.407039	0.132121
β1R-Ab	Insomnia	-0.02553	0.92804
β1R-Ab	Anxiety	0.356346	0.192339
β1R-Ab	Deflated mood	0.055546	0.844129
β1R-Ab	Arthromyalgia	0.268308	0.333599
β1R-Ab	Poor exercise tolerance	0.593682	0.019632
β1R-Ab	Irritability	0.060461	0.830512
β1R-Ab	Brain fog	0.234963	0.399252
β1R-Ab	Spike	-0.32913	0.25054
β1R-Ab	Nucleocapside	-0.30056	0.29643
β1R-Ab	Cortisol	-0.17234	0.539096
β1R-Ab	ACTH	-0.1312	0.654807
β1R-Ab	TSH	0.200746	0.473134
β2R-Ab	M1R-Ab	0.425036	0.114262
β2R-Ab	M2R-Ab	0.420309	0.118781
β2R-Ab	M3R-Ab	0.9833	5.29E-11
β2R-Ab	M4R-Ab	0.974815	7.49E-10

β2R-Ab	M5R-Ab	0.426811	0.112595
β2R-Ab	Total Antibodies	0.948119	7.7E-08
β2R-Ab	Median Antibodies	0.968178	3.37E-09
β2R-Ab	N Symptoms (total)	0.270222	0.330028
β2R-Ab	Dyspnoea	0.040411	0.886299
β2R-Ab	Cephalea	-0.02729	0.92309
β2R-Ab	Concentration/Memory deficit	0.224095	0.422026
β2R-Ab	Dysautonomia	0.362749	0.1839
β2R-Ab	Fatigue	0.377406	0.165499
β2R-Ab	Insomnia	-0.09363	0.739974
β2R-Ab	Anxiety	0.467083	0.079193
β2R-Ab	Deflated mood	0.076964	0.785142
β2R-Ab	Arthromyalgia	0.295123	0.285583
β2R-Ab	Poor exercise tolerance	0.552673	0.032627
β2R-Ab	Irritability	-0.02718	0.923413
β2R-Ab	Brain fog	0.239124	0.39071
β2R-Ab	Spike	-0.34714	0.223962
β2R-Ab	Nucleocapside	-0.3161	0.270899
β2R-Ab	Cortisol	-0.15285	0.586562
β2R-Ab	ACTH	-0.12593	0.667954
β2R-Ab	TSH	0.213842	0.444109
M1R-Ab	M2R-Ab	0.900276	4.79E-06
M1R-Ab	M3R-Ab	0.422095	0.117059
M1R-Ab	M4R-Ab	0.36724	0.178126
M1R-Ab	M5R-Ab	0.887852	9.96E-06
M1R-Ab	Total Antibodies	0.623276	0.01305
M1R-Ab	Median Antibodies	0.613367	0.015026
M1R-Ab	N Symptoms (total)	0.004437	0.987478
M1R-Ab	Dyspnoea	-0.37151	0.172746
M1R-Ab	Cephalea	-0.01402	0.960451
M1R-Ab	Concentration/Memory deficit	-0.11916	0.672299
M1R-Ab	Dysautonomia	-0.20474	0.464183
M1R-Ab	Fatigue	0.26452	0.34073
M1R-Ab	Insomnia	-0.06475	0.818661
M1R-Ab	Anxiety	0.098135	0.727882
M1R-Ab	Deflated mood	0.078157	0.781884
M1R-Ab	Arthromyalgia	0.354789	0.194428
M1R-Ab	Poor exercise tolerance	0.469455	0.077478
M1R-Ab	Irritability	0.131145	0.641301
M1R-Ab	Brain fog	-0.05431	0.847551
M1R-Ab	Spike	-0.09114	0.756662
M1R-Ab	Nucleocapside	-0.06616	0.822201
M1R-Ab	Cortisol	0.015628	0.955916
M1R-Ab	ACTH	0.202734	0.486987
M1R-Ab	TSH	0.245884	0.37704
M2R-Ab	M3R-Ab	0.461446	0.083379

M2R-Ab	M4R-Ab	0.419918	0.11916
M2R-Ab	M5R-Ab	0.959484	1.59E-08
M2R-Ab	Total Antibodies	0.664951	0.006835
M2R-Ab	Median Antibodies	0.625862	0.012569
M2R-Ab	N Symptoms (total)	-0.08617	0.760088
M2R-Ab	Dyspnoea	-0.4209	0.118212
M2R-Ab	Cephalea	-0.06013	0.831433
M2R-Ab	Concentration/Memory deficit	-0.15305	0.586047
M2R-Ab	Dysautonomia	-0.10022	0.722299
M2R-Ab	Fatigue	0.210937	0.450469
M2R-Ab	Insomnia	-0.03577	0.899299
M2R-Ab	Anxiety	0.060128	0.831433
M2R-Ab	Deflated mood	-0.06508	0.817758
M2R-Ab	Arthromyalgia	0.193562	0.489434
M2R-Ab	Poor exercise tolerance	0.430256	0.109411
M2R-Ab	Irritability	0.048552	0.863574
M2R-Ab	Brain fog	-0.16838	0.548592
M2R-Ab	Spike	-0.05215	0.859468
M2R-Ab	Nucleocapside	-0.12943	0.659211
M2R-Ab	Cortisol	0.112785	0.689008
M2R-Ab	ACTH	0.117984	0.687901
M2R-Ab	TSH	0.301862	0.274199
M3R-Ab	M4R-Ab	0.987264	9.18E-12
M3R-Ab	M5R-Ab	0.484158	0.067432
M3R-Ab	Total Antibodies	0.959911	1.48E-08
M3R-Ab	Median Antibodies	0.967745	3.68E-09
M3R-Ab	N Symptoms (total)	0.242141	0.384577
M3R-Ab	Dyspnoea	-0.03256	0.908284
M3R-Ab	Cephalea	-0.03256	0.908284
M3R-Ab	Concentration/Memory deficit	0.232591	0.404167
M3R-Ab	Dysautonomia	0.383815	0.157851
M3R-Ab	Fatigue	0.340177	0.214747
M3R-Ab	Insomnia	-0.04655	0.869146
M3R-Ab	Anxiety	0.493093	0.061807
M3R-Ab	Deflated mood	-0.03868	0.891139
M3R-Ab	Arthromyalgia	0.261411	0.346646
M3R-Ab	Poor exercise tolerance	0.53446	0.040121
M3R-Ab	Irritability	0.000879	0.997519
M3R-Ab	Brain fog	0.225052	0.419994
M3R-Ab	Spike	-0.35071	0.218922
M3R-Ab	Nucleocapside	-0.35504	0.212882
M3R-Ab	Cortisol	-0.12876	0.647425
M3R-Ab	ACTH	-0.11174	0.703729
M3R-Ab	TSH	0.206978	0.459207
M4R-Ab	M5R-Ab	0.44364	0.097631
M4R-Ab	Total Antibodies	0.947137	8.68E-08

M4R-Ab	Median Antibodies	0.952386	4.45E-08
M4R-Ab	N Symptoms (total)	0.249148	0.370534
M4R-Ab	Dyspnoea	-0.02031	0.942726
M4R-Ab	Cephalea	0.034817	0.901961
M4R-Ab	Concentration/Memory deficit	0.246624	0.37556
M4R-Ab	Dysautonomia	0.33404	0.223668
M4R-Ab	Fatigue	0.330947	0.22825
M4R-Ab	Insomnia	-0.03071	0.91348
M4R-Ab	Anxiety	0.472938	0.075007
M4R-Ab	Deflated mood	-0.05483	0.846109
M4R-Ab	Arthromyalgia	0.175891	0.53063
M4R-Ab	Poor exercise tolerance	0.522091	0.045894
M4R-Ab	Irritability	0.027416	0.922736
M4R-Ab	Brain fog	0.246746	0.375317
M4R-Ab	Spike	-0.2983	0.30024
M4R-Ab	Nucleocapside	-0.33828	0.236805
M4R-Ab	Cortisol	-0.14661	0.602101
M4R-Ab	ACTH	-0.15409	0.598908
M4R-Ab	TSH	0.169387	0.546173
M5R-Ab	Total Antibodies	0.68389	0.004931
M5R-Ab	Median Antibodies	0.626369	0.012476
M5R-Ab	N Symptoms (total)	0.023289	0.934343
M5R-Ab	Dyspnoea	-0.42161	0.117525
M5R-Ab	Cephalea	0.05394	0.848587
M5R-Ab	Concentration/Memory deficit	-0.05394	0.848587
M5R-Ab	Dysautonomia	-0.1272	0.651441
M5R-Ab	Fatigue	0.260979	0.347473
M5R-Ab	Insomnia	0.116942	0.678105
M5R-Ab	Anxiety	0.080359	0.775882
M5R-Ab	Deflated mood	-0.10526	0.70888
M5R-Ab	Arthromyalgia	0.232612	0.404123
M5R-Ab	Poor exercise tolerance	0.496366	0.059834
M5R-Ab	Irritability	0.135014	0.631406
M5R-Ab	Brain fog	0.025796	0.92729
M5R-Ab	Spike	-0.0638	0.828458
M5R-Ab	Nucleocapside	-0.10862	0.711663
M5R-Ab	Cortisol	-0.05546	0.84437
M5R-Ab	ACTH	0.109438	0.709577
M5R-Ab	TSH	0.269891	0.330646
Total Antibodies	Median Antibodies	0.991321	7.66E-13
Total Antibodies	N Symptoms (total)	0.22095	0.428739
Total Antibodies	Dyspnoea	-0.11679	0.67849
Total Antibodies	Cephalea	0.028109	0.92079
Total Antibodies	Concentration/Memory deficit	0.164292	0.558481
Total Antibodies	Dysautonomia	0.24793	0.372954
Total Antibodies	Fatigue	0.382781	0.159069

Total Antibodies	Insomnia	-0.01305	0.963186
Total Antibodies	Anxiety	0.396734	0.143158
Total Antibodies	Deflated mood	-0.01739	0.950955
Total Antibodies	Arthromyalgia	0.274693	0.321773
Total Antibodies	Poor exercise tolerance	0.611188	0.015491
Total Antibodies	Irritability	0.019362	0.945397
Total Antibodies	Brain fog	0.199538	0.475857
Total Antibodies	Spike	-0.29582	0.304485
Total Antibodies	Nucleocapside	-0.31017	0.280482
Total Antibodies	Cortisol	-0.14828	0.597924
Total Antibodies	ACTH	-0.10152	0.729841
Total Antibodies	TSH	0.251532	0.365823
Median Antibodies	N Symptoms (total)	0.214106	0.443533
Median Antibodies	Dyspnoea	-0.08805	0.755018
Median Antibodies	Cephalea	-0.02549	0.928158
Median Antibodies	Concentration/Memory deficit	0.161035	0.566414
Median Antibodies	Dysautonomia	0.267993	0.33419
Median Antibodies	Fatigue	0.372967	0.170938
Median Antibodies	Insomnia	-0.0903	0.748941
Median Antibodies	Anxiety	0.415909	0.123096
Median Antibodies	Deflated mood	0.033936	0.904433
Median Antibodies	Arthromyalgia	0.304337	0.270088
Median Antibodies	Poor exercise tolerance	0.588608	0.020978
Median Antibodies	Irritability	0.020799	0.941349
Median Antibodies	Brain fog	0.18172	0.516873
Median Antibodies	Spike	-0.29963	0.297996
Median Antibodies	Nucleocapside	-0.30282	0.292618
Median Antibodies	Cortisol	-0.10678	0.704859
Median Antibodies	ACTH	-0.06726	0.819294
Median Antibodies	TSH	0.264619	0.340542
N Symptoms (total)	Dyspnoea	0.66368	0.006981
N Symptoms (total)	Cephalea	0.602417	0.017471
N Symptoms (total)	Concentration/Memory deficit	0.929152	5.57E-07
N Symptoms (total)	Dysautonomia	0.441906	0.099104
N Symptoms (total)	Fatigue	0.896874	5.91E-06
N Symptoms (total)	Insomnia	0.495181	0.060543
N Symptoms (total)	Anxiety	0.479892	0.070244
N Symptoms (total)	Deflated mood	0.461173	0.083585
N Symptoms (total)	Arthromyalgia	0.654346	0.008129
N Symptoms (total)	Poor exercise tolerance	0.683822	0.004937
N Symptoms (total)	Irritability	0.519061	0.047397
N Symptoms (total)	Brain fog	0.779557	0.000611
N Symptoms (total)	Spike	-0.1558	0.594809
N Symptoms (total)	Nucleocapside	0.179961	0.538136
N Symptoms (total)	Cortisol	-0.7526	0.001205
N Symptoms (total)	ACTH	-0.5166	0.058568

N Symptoms (total)	TSH	0.032499	0.908464
Dyspnoea	Cephalea	0.4	0.139595
Dyspnoea	Concentration/Memory deficit	0.5	0.057699
Dyspnoea	Dysautonomia	0.533002	0.040772
Dyspnoea	Fatigue	0.426401	0.112979
Dyspnoea	Insomnia	-2.1E-17	1
Dyspnoea	Anxiety	0.4	0.139595
Dyspnoea	Deflated mood	0.472456	0.075346
Dyspnoea	Arthromyalgia	0.288675	0.296732
Dyspnoea	Poor exercise tolerance	0.288675	0.296732
Dyspnoea	Irritability	-0.09449	0.737652
Dyspnoea	Brain fog	0.472456	0.075346
Dyspnoea	Spike	0.139427	0.634515
Dyspnoea	Nucleocapside	0.239366	0.409822
Dyspnoea	Cortisol	-0.4809	0.069571
Dyspnoea	ACTH	-0.53	0.051246
Dyspnoea	TSH	0.089804	0.750274
Cephalea	Concentration/Memory deficit	0.5	0.057699
Cephalea	Dysautonomia	-0.1066	0.705338
Cephalea	Fatigue	0.426401	0.112979
Cephalea	Insomnia	0.57735	0.024215
Cephalea	Anxiety	0.1	0.722897
Cephalea	Deflated mood	-0.09449	0.737652
Cephalea	Arthromyalgia	1.39E-17	1
Cephalea	Poor exercise tolerance	0.57735	0.024215
Cephalea	Irritability	0.188982	0.499964
Cephalea	Brain fog	0.472456	0.075346
Cephalea	Spike	0.351001	0.218508
Cephalea	Nucleocapside	0.333647	0.2437
Cephalea	Cortisol	-0.66692	0.006613
Cephalea	ACTH	-0.32159	0.262193
Cephalea	TSH	-0.3651	0.180857
Concentration/Memory deficit	Dysautonomia	0.426401	0.112979
Concentration/Memory deficit	Fatigue	0.852803	5.34E-05
Concentration/Memory deficit	Insomnia	0.57735	0.024215
Concentration/Memory deficit	Anxiety	0.5	0.057699
Concentration/Memory deficit	Deflated mood	0.377964	0.164823
Concentration/Memory deficit	Arthromyalgia	0.57735	0.024215
Concentration/Memory deficit	Poor exercise tolerance	0.57735	0.024215

Concentration/Memory deficit	Irritability	0.661438	0.007244
Concentration/Memory deficit	Brain fog	0.661438	0.007244
Concentration/Memory deficit	Spike	-0.19186	0.511115
Concentration/Memory deficit	Nucleocapside	0.173346	0.553418
Concentration/Memory deficit	Cortisol	-0.59803	0.018534
Concentration/Memory deficit	ACTH	-0.44518	0.110682
Concentration/Memory deficit	TSH	-0.01693	0.952247
Dysautonomia	Fatigue	0.363636	0.182749
Dysautonomia	Insomnia	-0.18464	0.51005
Dysautonomia	Anxiety	0.533002	0.040772
Dysautonomia	Deflated mood	0.040291	0.886633
Dysautonomia	Arthromyalgia	0.492366	0.062252
Dysautonomia	Poor exercise tolerance	0.184637	0.51005
Dysautonomia	Irritability	0.040291	0.886633
Dysautonomia	Brain fog	0.342475	0.211467
Dysautonomia	Spike	-0.47338	0.087314
Dysautonomia	Nucleocapside	-0.42651	0.128304
Dysautonomia	Cortisol	-0.18009	0.520714
Dysautonomia	ACTH	-0.48559	0.078366
Dysautonomia	TSH	0.058223	0.836706
Fatigue	Insomnia	0.492366	0.062252
Fatigue	Anxiety	0.426401	0.112979
Fatigue	Deflated mood	0.564076	0.028504
Fatigue	Arthromyalgia	0.738549	0.001663
Fatigue	Poor exercise tolerance	0.738549	0.001663
Fatigue	Irritability	0.564076	0.028504
Fatigue	Brain fog	0.564076	0.028504
Fatigue	Spike	-0.27296	0.345069
Fatigue	Nucleocapside	0.111964	0.703147
Fatigue	Cortisol	-0.58227	0.022758
Fatigue	ACTH	-0.46533	0.093598
Fatigue	TSH	0.123038	0.66222
Insomnia	Anxiety	-4.2E-17	1
Insomnia	Deflated mood	0.054554	0.84688
Insomnia	Arthromyalgia	0.111111	0.693416
Insomnia	Poor exercise tolerance	0.388889	0.151966
Insomnia	Irritability	0.327327	0.233688
Insomnia	Brain fog	0.327327	0.233688
Insomnia	Spike	-0.12022	0.682275
Insomnia	Nucleocapside	0.254194	0.380505

Insomnia	Cortisol	-0.56803	0.027173
Insomnia	ACTH	-0.30193	0.294112
Insomnia	TSH	-0.00297	0.991605
Anxiety	Deflated mood	0.188982	0.499964
Anxiety	Arthromyalgia	0.288675	0.296732
Anxiety	Poor exercise tolerance	0.288675	0.296732
Anxiety	Irritability	0.188982	0.499964
Anxiety	Brain fog	0.188982	0.499964
Anxiety	Spike	-0.10838	0.712286
Anxiety	Nucleocapside	-0.05719	0.846016
Anxiety	Cortisol	-0.12539	0.65612
Anxiety	ACTH	-0.37271	0.189371
Anxiety	TSH	0.036805	0.896392
Deflated mood	Arthromyalgia	0.49099	0.063099
Deflated mood	Poor exercise tolerance	0.218218	0.434614
Deflated mood	Irritability	0.196429	0.482899
Deflated mood	Brain fog	0.196429	0.482899
Deflated mood	Spike	-0.00243	0.993418
Deflated mood	Nucleocapside	0.414455	0.140645
Deflated mood	Cortisol	-0.18072	0.519218
Deflated mood	ACTH	-0.18329	0.530507
Deflated mood	TSH	0.4873	0.065413
Arthromyalgia	Poor exercise tolerance	0.444444	0.096952
Arthromyalgia	Irritability	0.49099	0.063099
Arthromyalgia	Brain fog	0.49099	0.063099
Arthromyalgia	Spike	-0.52509	0.05385
Arthromyalgia	Nucleocapside	-0.22207	0.445443
Arthromyalgia	Cortisol	-0.42642	0.112964
Arthromyalgia	ACTH	-0.17078	0.559385
Arthromyalgia	TSH	0.164469	0.558051
Poor exercise tolerance	Irritability	0.218218	0.434614
Poor exercise tolerance	Brain fog	0.49099	0.063099
Poor exercise tolerance	Spike	-0.00281	0.9924
Poor exercise tolerance	Nucleocapside	0.28851	0.317144
Poor exercise tolerance	Cortisol	-0.47017	0.076964
Poor exercise tolerance	ACTH	-0.13847	0.636869
Poor exercise tolerance	TSH	0.056098	0.842597
Irritability	Brain fog	0.464286	0.081251
Irritability	Spike	-0.18799	0.519833
Irritability	Nucleocapside	0.060847	0.836297
Irritability	Cortisol	-0.25103	0.366809
Irritability	ACTH	-0.08811	0.764537
Irritability	TSH	-0.1533	0.58544
Brain fog	Spike	-0.23713	0.414348
Brain fog	Nucleocapside	0.070418	0.810942
Brain fog	Cortisol	-0.78226	0.000568

Brain fog	ACTH	-0.40132	0.154962
Brain fog	TSH	0.053279	0.850423
Spike	Nucleocapside	0.530916	0.05077
Spike	Cortisol	0.268657	0.353025
Spike	ACTH	0.276752	0.360001
Spike	TSH	-0.1399	0.633344
Nucleocapside	Cortisol	-0.05763	0.844855
Nucleocapside	ACTH	0.278124	0.357527
Nucleocapside	TSH	0.094417	0.748168
Cortisol	ACTH	0.421424	0.13342
Cortisol	TSH	0.034005	0.904238
ACTH	TSH	-0.231	0.426869
